# Confounding Factors Influencing Amyloid Beta Concentration in Cerebrospinal Fluid

**DOI:** 10.4061/2010/986310

**Published:** 2010-07-15

**Authors:** Maria Bjerke, Erik Portelius, Lennart Minthon, Anders Wallin, Henrik Anckarsäter, Rolf Anckarsäter, Niels Andreasen, Henrik Zetterberg, Ulf Andreasson, Kaj Blennow

**Affiliations:** ^1^Department of Psychiatry and Neurochemistry, Institute of Neuroscience and Physiology, The Sahlgrenska Academy at University of Gothenburg, 431 80 Mölndal, Sweden; ^2^Department of Clinical Sciences Malmö, Clinical Memory Research Unit, Lund University, 205 02 Malmö, Sweden; ^3^Forensic Psychiatry, Institute of Neuroscience and Psychology, The Sahlgrenska Academy at University of Gothenburg, 422 50 Gothenburg, Sweden; ^4^Department of Neurobiology, Karolinska Institute, Caring Sciences and Society, Karolinska University Hospital, Huddinge, 141 86 Stockholm, Sweden; ^5^Department of Geriatric Medicine, Karolinska Institute, Memory Clinic, M51, Karolinska University Hospital, Huddinge, 141 86 Stockholm, Sweden

## Abstract

*Background*. Patients afflicted with Alzheimer's disease (AD) exhibit a decrease in the cerebrospinal fluid (CSF) concentration of the 42 amino acid form of *β*-amyloid (A*β*_42_). However, a high discrepancy between different centers in measured A*β*_42_ levels reduces the utility of this biomarker as a diagnostic tool and in monitoring the effect of disease modifying drugs. Preanalytical and analytical confounding factors were examined with respect to their effect on the measured A*β*_42_ level. *Methods*. Aliquots of CSF samples were either treated differently prior to A*β*_42_ measurement or analyzed using different commercially available xMAP or ELISA assays. *Results*. Confounding factors affecting CSF A*β*_42_ levels were storage in different types of test tubes, dilution with detergent-containing buffer, plasma contamination, heat treatment, and the origin of the immunoassays used for quantification. *Conclusion*. In order to conduct multicenter studies, a standardized protocol to minimize preanalytical and analytical confounding factors is warranted.

## 1. Introduction

By the year of 2000, it was estimated that more than 25 million people suffered from dementia, with Alzheimer's disease (AD) being the most common subtype accounting for around 50 percent of all cases [1, 2]. Histopathological hallmarks of AD include intracellular neurofibrillary tangles composed of tau protein and extracellular deposits of neurotoxic *β*-amyloid (A*β*) visualized as amyloid plaques [3–5]. The cerebrospinal fluid (CSF) concentrations of A*β* peptides in combination with the tau protein and its hyperphosphorylated forms have been found to support the clinical diagnosis of AD [6]. Not only do these biomarkers fulfill the criteria for an ideal diagnostic test according to the guidelines of the Working group on molecular and biochemical markers of Alzheimer's disease [7], but A*β* have also been suggested to be a driving force in the disease process. The amyloid cascade hypothesis proposes that an imbalance in A*β* production and clearance leads to an increase in A*β* load and that this initiates taupathology and neuronal degeneration which ultimately causes dementia [8]. The hypothesis is derived from cases affected by rare familial forms of AD wherein mutations in the amyloid precursor protein (*APP*) gene or in the presenilin-encoding (*PSEN1 *and *PSEN2) *genes, which are involved in metabolizing the APP protein, invariably lead to AD pathology. The 42 amino acid long form of A*β* (A*β*_42_) has also proven to be the best established CSF biomarker for amyloid pathology in the brain. A*β* has therefore become the primary target of many clinical trials in their search for novel treatment strategies as well as a core biomarker candidate for monitoring disease-modifying effects [9]. 

 Recently a large multicenter study assessed the diagnostic value of the 42 amino acid long form of A*β* (A*β*_42_), total tau (T-tau), and tau phosphorylated at threonine 181 (P-tau_181_) in identifying subjects with incipient AD among patients with mild cognitive impairment (MCI) and they were found to provide good accuracy [10]. The neuropathologic correlates distinguishing those of the MCI patients thought to await in the precedent stage of clinical overt AD [11], are seemingly reflected by these biomarkers. Although the biomarkers show reasonable accuracy to discriminate controls from AD patients as well as prodromal AD in MCI patients [12–15], it has been shown in population-based studies that healthy elderly people who later develop AD have reductions in CSF A*β*_42_ levels while tau levels are normal [16, 17]. However, there is a high discrepancy in the reported concentrations of these biomarkers [18] leading to different cut-off values between different centers with the highest variability shown for A*β*_42_ [10]. This type of between-center variability in analytical results may be due to differences in preanalytical procedures for CSF collection and sample processing, analytical procedures and techniques, and to batch-to-batch variation for the immunoassay kits. It has been suggested that preanalytical confounding factors such as CSF collection, storage, and adsorption to tube-walls contribute to the highest magnitude of errors [19]. This paper aims at assessing these preanalytical confounding factors together with other factors such as blood contamination, blood-brain barrier dysfunction, sample pretreatment and differences in assay performance regarding the impact on measured A*β*_42_ levels.

## 2. Material and Methods

### 2.1. CSF and Plasma Samples

All CSF samples were obtained by lumbar puncture (LP) between the L3/L4 and L4/L5 intervertebral space. Except when otherwise noted, a volume of 10–12 mL CSF was collected in polypropylene tubes followed by centrifugation (2000 × g, 10 min, 4°C) and storage in smaller aliquots at −80°C. Plasma was obtained by the centrifugation (2 500 × g, 10 min, 4°C) of whole blood in EDTA tubes (BD, art. nr. 367864). The plasma was aliquoted into polypropylene tubes and stored at −80°C pending analysis. All samples were thawed at room temperature (RT), if nothing else is declared. The samples used for evaluation of confounding factors were aliquots from samples sent for routine diagnostic purposes. All samples were decoded so that no information could be linked to an individual patient.

### 2.2. Subjects

Two case control studies assessed the differences in CSF A*β*_42_ levels due to differences in pretreatment, the first study comprised 15 AD and 15 control samples and the second comprised 20 AD and 20 control samples. The patients who received the diagnosis of AD fulfilled the DSM-III-R criteria of dementia [20] and the criteria of probable AD defined by NINCDS-ADRDA (National Institute of Neurological and Communicative Disorders—Stroke/Alzheimer's Disease and Related Disorders Association) [21]. Healthy controls were mainly recruited from senior citizens' organizations, while a few were spouses of study patients. Controls were not included if they had a history or subjective or objective signs of a cognitive disorder. 

 The study was conducted according to the provisions of the Helsinki Declaration and was approved by the ethics committee of the Universities of Gothenburg and Lund and the Karolinska Institute, Sweden.

### 2.3. CSF Analysis

Unless otherwise stated, the CSF A*β*_42_ concentrations were obtained using the Innogenetics NV INNO-BIA xMAP technology (INNO-BIA AlzBio3) [22]. For practical reasons some tests were analyzed using the established Innogenetics enzyme-linked immunosorbent assay (ELISA) (INNOTEST *β*-amyloid_1–42_) [23], using a slightly modified protocol [24], which has previously been shown to correlate well with the Innogenetics AlzBio3 assay [22]. Since preanalytical factors were to be assessed and not absolute A*β*_42_ levels, using different assays should pose no problems. In a case control study, these assays were compared with four other commercially available A*β*_42_ immunoassays from Innogenetics (INNO-BIA plasma A*β* forms) [25], Meso-Scale Discovery (MSD 96-Well MULTI-SPOT, Human/Rodent (4G8) Abeta Triplex Ultra-Sensitive Assay) [26], and The Genetics Company (hAmyloid *β*42 ELISA) [27, 28], to evaluate their performance in discriminating between AD patients and healthy controls. All analyses were performed according to manufacturers' instructions; however, the Innogenetics ELISA was also performed by replacing the detection antibody with the 4G8 monoclonal antibody. The capture and detection antibodies for each assay are summarized in Table 1. Whenever practically possible the samples for a specific experiment were run on the same plate in order to eliminate errors caused by interassay variability.

### 2.4. Statistical Analysis

Since several variables were found to be skewed the nonparametric Friedman's or Wilcoxon tests were used for pairwise comparisons while the Mann-Whitney *U*-test was employed for unpaired comparisons. The data is presented as median and percentiles (5th and 95th). Correlation analyses were performed using the Spearman correlation coefficient (rho). Receiver operating characteristic (ROC) analyses were performed to evaluate the discrimination power of the different assays using the area under the curve (AUC). SPSS 15.0 was employed for all univariate analyses.

### 2.5. Sample Collection and Assessment of Confounding Factors 

#### 2.5.1. Adsorption to Test Tubes and Lumbar Catheter Walls

Freshly collected CSF from ten different subjects was tapped into glass (Schott, art. nr. 2317103), polypropylene (Sarstedt, art. nr. 60.549), and polystyrene (Sarstedt, art. nr. 55.476) tubes. The tubes were incubated at RT for one hour on a Boule mixer and thereafter stored in a freezer (−80°C) pending analysis. 

 Six samples of CSF were divided into three aliquots of which one was run through a lumbar catheter (Braun-Perifix, art. nr. 4513150), one was run through a lumbar pressure meter catheter (Mediplast, art. nr. 6061650008), while the third aliquot was analyzed without any pretreatment.

#### 2.5.2. Incubation, Storage, and Collection of CSF

To test whether A*β*_42_ is sensitive to handling at RT, eight freshly collected samples, within 3 hours after withdrawal, were divided into two aliquots. The first aliquot was analyzed immediately, while the other was analyzed after 24 hours of incubation at RT. Six control samples were set on each plate to adjust the levels according to the interassay variability. 

 In order to test if A*β*_42_ is sensitive to freezing eight samples of CSF were collected freshly and divided into two aliquots. The first aliquot was analyzed immediately while the second was stored at −80°C for at least one week pending analysis. Two control samples, each analyzed in two duplicates, were used to adjust for interassay variability. The samples were analyzed using Innogenetics ELISA. 

 Data on the long-term storage stability of A*β*_42_ in CSF at −80°C were retrieved from repeated analyses (*N* = 214) of an internal quality control sample (aliquots of a large CSF pool) during 26 months. The control sample was analyzed in duplicates at each occasion using the Innogenetics ELISA. 

 To test for the impact of incubation at RT in combination with freezing, twelve samples of CSF were collected freshly and divided into seven aliquots of which one was immediately frozen. The remaining six aliquots were stored at either +4°C or RT for four hours, 24 hours or three days and thereafter frozen pending simultaneous analysis using Innogenetics ELISA. 

 To test for the sensitivity of different processes of freezing, different freezing temperatures, and different thawing conditions freshly collected CSF from ten subjects was divided into eight aliquots and analyzed. Two aliquots were tested for the process of freezing; one was frozen on dry ice/ethanol and thereafter stored at −80°C for three days while the other was immediately stored at −80°C. Three aliquots were frozen at −20°C at stable temperature, −20°C in an auto-defrosting freezer, or −80°C for three days. Three aliquots were stored at −80°C for three days and thereafter tested for thawing in a fridge (+4°C), at RT and in a water bath at +20°C. 

 To test whether the initial LP conditions influence the biomarker level, freshly collected CSF from nine subjects was either collected at RT or in tubes placed on ice. The samples were left to incubate for three hours and thereafter frozen at −80°C.

#### 2.5.3. *A* *β* Spinal Chord Gradient

The A*β*_42_ CSF spinal chord gradient was assessed by sequentially withdrawing four fractions of 10 mL CSF from seven patients; fraction 1 (0–10 mL), 2 (11–20 mL), 3 (21–30 mL) and 4 (31–40 mL).

#### 2.5.4. Contamination of Blood and Blood-Brain Barrier Dysfunction

To examine if blood contamination influence CSF biomarkers a blood sample was diluted by water and freeze-thawn in dry ice/ethanol in order to lyse erythrocytes. Serially diluted blood equivalent to an erythrocyte level of 200, 1000 and 5000 /*μ*L (0.004–0.1% v/v) was then added to 10 different CSF samples (95% v/v) and compared with the corresponding water-diluted CSF (95% v/v). The samples were analyzed using Innogenetics ELISA. 

 To test the influence of blood-brain barrier (BBB) dysfunction on CSF biomarkers a plasma sample was serially diluted by water to an albumin level corresponding of approximately 0.25, 0.50, 1.0 and 2.0 g/L (0.625–5% v/v) when added to 8 CSF samples (90% v/v) with an albumin level of approximately 0.20 g/L. The samples were compared with corresponding water-diluted CSF (90% v/v). This series represents different degrees of BBB permeability with a CSF/serum albumin ratio of 5, 11, 18, 30 and 55, respectively (5 equals the CSF baseline level ((0.20 g/L / 40 g/L (serum albumin)) × 1000)). The samples were analyzed using the Innogenetics ELISA.

#### 2.5.5. The Influence of an Overnight Fast on Plasma *A* *β*_42_ Levels

Blood was withdrawn at three different occasions from nine cognitively healthy subjects to test whether fasting or subsequent food intake influences the plasma baseline A*β*_42_ levels. Plasma was preferred as opposed to CSF due to the inconvenience of repeated fluid sampling by LP on healthy subjects. The baseline blood sample was withdrawn at nine o'clock in the morning following a nonstandardized breakfast, the follow-up sample after three weeks at the same time in the morning following an overnight fast and the postprandial sample one hour after a standardized breakfast the same day. The samples were analyzed using Innogenetics INNO-BIA plasma A*β* forms.

#### 2.5.6. Diurnal Variability

CSF from 14 psychiatrically and neurologically healthy subjects undergoing knee prothesis surgery was serially collected by LP with an 18-Gauge Portex epidural needle at baseline, after four to six hours (mean 5.3 hours) and after 24 hours, for further details on this procedure see Anckarsäter et al. [29]. The samples were immediately stored at −80°C pending analysis.

#### 2.5.7. Centrifugation and Heat Denaturation

CSF from ten subjects was divided into three aliquots each of which one was analyzed without any pretreatment. The other two aliquots were centrifuged for ten minutes (2000 × g), at RT or at +4°C to evaluate the effect of A*β* precipitation during centrifugation. Furthermore, 18 samples of CSF were divided into two aliquots of which one was boiled at 100°C in a heating block for ten minutes. Both samples were thereafter centrifuged at +4°C for ten minutes (2000 × g). 

 CSF from 15 AD patients and 15 controls were divided into two aliquots of which one was preincubated for 15 minutes at 100°C in a heating block. Both aliquots were centrifuged (2000 × *g*) at +4°C for ten minutes and analyzed using the Innogenetics ELISA.

#### 2.5.8. Sample Pretreatment Affecting Assay Analyses

A fresh CSF sample was used to assess the impact of incubation at RT on changes in pH. The baseline pH was measured within 30 minutes from LP. 

 A CSF pool was serially diluted (50%, 25%, 6.25% and 3.125% v/v) in different buffers as well as in the provided assay buffer. Different buffer concentrations (10, 50 and 100 mM phosphate buffered saline (PBS) (pH 7.4)), and different buffer substances (PBS, Tris and HEPES (pH 7.4)), were tested for the effect on the measured A*β*_42_ concentration. Furthermore, the effect on assay performance at different pH was evaluated in 50 mM Tris (pH 7.4, 8, and 9). The addition of 0.1% v/v Tween 20, 0.05% v/v Triton X100 and 0.5 mg/mL BSA to 50 mM PBS (pH7.4) was used to further assess the improvement of A*β*_42_ detection.

#### 2.5.9. Epitope Masking and/or Assay Specific Variability

Divergences in measured A*β* concentration levels between different commercially available A*β*_42_ assays were evaluated and the assays were compared with respect to how well they perform in discriminating between AD patients and healthy controls. CSF from patients with AD (*n* = 20) and healthy controls (*n* = 20) was analyzed using assays from Innogenetics (ELISA, Abeta forms, and AlzBio3), Meso-Scale Discovery (Triplex), and The Genetics (ELISA). The commercial Innogenetics ELISA was also tested by replacing the detection antibody with the monoclonal antibody 4G8. Neat and threefold diluted (0.05% Tween 20 in PBS) CSF samples were analyzed according to the instructions from the manufacturers.

## 3. Results and Discussion

### 3.1. Adsorption to Test Tubes and Lumbar Catheter Walls

The A*β*_42_ levels were significantly altered by storage in different test tubes. The A*β*_42_ levels significantly decreased when CSF was stored in polystyrene tubes (208 ng/L (126–467)) compared with polypropylene (271 ng/L (152–478)), as previously shown by others [23, 30, 31], rendering a significantly decreased A*β*_42_ level of as much as 35% (mean decrease 19%; *P* = .002). The A*β*_42_ level also decreased when CSF was stored in glass tubes compared with polypropylene, however it did not reach statistical significance. Some of the previously reported differences in absolute values of A*β*_42_ might thus be due to adsorption to different test tubes probably caused by the hydrophobic nature of this analyte. In consequence, standardization of collection tubes is necessary in order to be able to compare absolute concentration values among different centers. 

 Adhesion of A*β*_42_ to the lumbar catheter walls during LP might also render a difference in analyte concentration and would urge for standardization. However, two different catheters were tested of which none significantly altered the concentration of A*β*_42_ as compared with the baseline level.

### 3.2. Incubation, Storage and Collection of CSF

It is essential for reliable biochemical analysis that the stability of a biomarker is thoroughly investigated in order to implement the appropriate preanalytical handling. Eight CSF samples were analyzed within three hours from withdrawal and after 24 hours of incubation at RT. No significant difference between the paired samples was detected in the A*β*_42_ levels suggesting that this biomarker is stable when left for at least a day at RT. Furthermore, no significant alteration in the level of A*β*_42_, as previously described [32], was found between fresh CSF and CSF that had undergone one freeze/thaw cycle. Nor were there any significant differences between the baseline A*β*_42_ levels of freshly frozen samples and samples frozen after incubation at RT or at +4°C for four hours, 24 hours or three days. These results are in contrast to one study, which found A*β*_42_ to be decrease by 20% after two days incubation at RT, while no difference was found when comparing fresh CSF to frozen/thawed CSF [33]. However, the fresh CSF had been incubated at RT during two days, which would mean that the level of A*β*_42_ implemented as a baseline value was decreased and thus also the A*β*_42_ level in the frozen CSF. Furthermore, the study had a very limited sample size which may contribute to the divergent results. Another study showed, contradictory to ours, that the A*β*_42_ concentration was increased after 24 hours of incubation at RT [34]. However, the CSF was not centrifuged prior to incubation which seems to affect the outcome, see below. 

**Figure 1:**
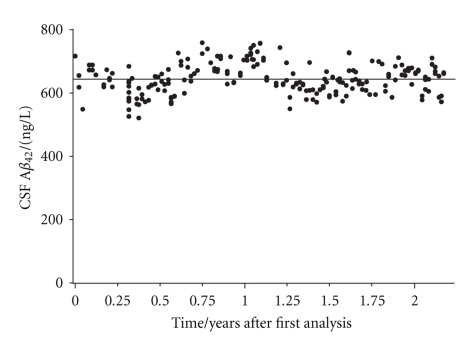
The mean A*β*_42_ variation in CSF during 26 months. An internal quality control sample was run on 214 different occasions rendering a mean ± SD value (—) of 643 ± 48 ng/L and a CV of 7.5%.

 The storage stability of CSF A*β*_42_ at −80°C was assessed through an internal quality control sample which was analyzed on a weekly basis during a time period of 26 months. The coefficient of variation (CV) on 214 different runs was 7.5%, which is less than the inter assay CV (7.7%) reported by the manufacturer (Innogenetics NV) and consequently signify the storage stability of A*β*_42_ during the accounted time period (Figure 1). The storage stability of A*β*_42_ in CSF during shorter time periods has previously been reported further supporting this finding [35]. 

 No significant changes accounting for larger differences than the intraindividual assay CV (3.8%) was seen for neither of the various routes for freezing, the different storage temperatures, nor the diverse thawing procedures. Moreover, no difference in A*β*_42_ concentration was found when comparing CSF collected and incubated in tubes placed on ice during the LP procedure compared with CSF collected and incubated at RT. This is an indication of the A*β*_42_ stability and supports the use of this peptide as a CSF biomarker. However, other proteins may be sensitive to storage at −20°C. Storage at −20°C causes, for instance, a truncation in Cystatin C revealed by a peptide artifact identified by gel electrophoresis and mass spectrometry [36, 37]. Therefore, it is recommendable to store CSF samples at −80°C as a precaution to possible future analyses. 

### 3.3. A*β* Spinal Chord Gradient

Since CSF proteins originating from brain cells may have a decreasing rostro-caudal concentration gradient, while proteins released from the leptomeninges and blood derived proteins have a lower ventricular than lumbar CSF concentration, withdrawal of different CSF volumes might affect the outcome of biochemical analysis [38]. By withdrawing a small volume of CSF the biochemical composition might only reflect that of the lumbar dural sac and the withdrawal of a too large volume might influence the analysis as to increase the concentration of a brain specific protein. No spinal chord gradient was detected for A*β*_42_ when successively withdrawing four 10 mL portions CSF; that is, the four portions did not significantly differ in their A*β*_42_ level. Although there was no gradient for CSF A*β*_42_ along the spinal chord, it is still recommended to take a standardized volume of CSF at LP since other proteins such as albumin [39] and especially neurotransmitters [40] will be affected.

### 3.4. Contamination of Blood and Blood-Brain Barrier Dysfunction

**Figure 2:**
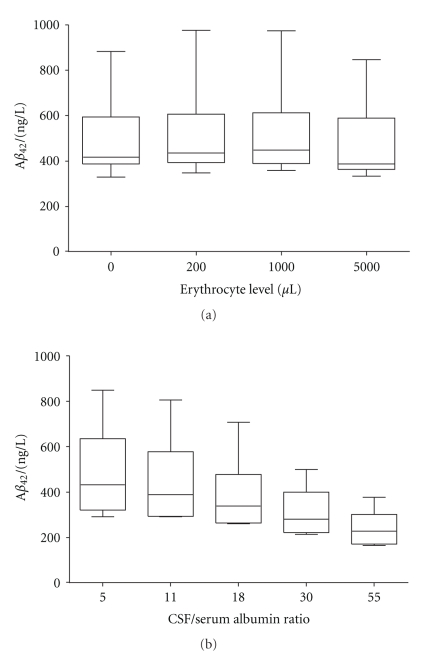
Boxplots with whiskers representing minutes and max values. (a) The effect of blood contamination on CSF A*β*_42_ levels. The addition of blood to CSF representing an erythrocyte level of 200, 1000 and 5000 /*μ*L of blood did not affect the A*β*_42_ level compared with neat CSF. (b) The effect a blood-brain barrier dysfunction on A*β*_42_ levels. Plasma was added to CSF at a concentration representing a CSF/serum albumin ratio of 11, 18, 30 and 55. The A*β*_42_ concentration was significantly decreased (*P* < .01) at all added plasma concentrations.

It is not uncommon that the CSF gets contaminated by blood during the LP procedure [41]. Since the concentration of proteins in CSF is about 0.5% that of blood [42] only a minor leakage could lead to an altered biomarker profile. Furthermore, blood contamination of CSF could lead to an increase in protein degradation already visible after 6 hours of incubation [43]. Therefore, it is important that contaminated CSF is discarded and that CSF is centrifuged as soon as possible after LP to get rid of contaminants invisible to the eye. Consequently, the addition of 0.1%, 0.02% and 0.004% (corresponding to 5000, 1000 and 200 erythrocytes/*μ*L) of blood to CSF should provide reliable information on the impact of contamination, unnoticeable to the eye, on the A*β*_42_ levels. However, no significant changes accounting for larger differences than the intraindividual assay CV was seen when comparing CSF contaminated with blood to neat CSF (Figure 2(a)). 

 Neat CSF was compared with CSF with added plasma, corresponding to a CSF/serum albumin ratio of 5, 11, 18, 30 and 55, (i.e., a range from normal to pathological blood-CSF barrier function), and the A*β*_42_ concentration in the diluted CSF was significantly (*P* = .008) decreased by as much as 49% (228 ng/L (165–378)) compared with the neat CSF A*β*_42_ concentration (433 ng/L (291–851)) (Figure 2(b)). One explanation to the decrease might be a high concentration of several proteins that bind A*β* in plasma, such as albumin [44], *α*2-macroglobulin [45] and low-density receptor related protein-1 [46], and it might explain the fact that numerous studies have found no correlation between CSF and plasma levels of A*β* biomarkers [32, 47], for review see [48]. It may be important to consider the albumin ratio when evaluating the concentration of A*β*_42_ in CSF in disorders with severe impairment of the BBB, such as acute meningitis [49], due to the impact of plasma on the measured CSF A*β* levels.

### 3.5. The Influence of an Overnight Fast on Plasma *A* *β*_42_ Levels

Even though the absolute CSF A*β*_42_ values have diverged among different centers the decreased A*β*_42_ levels in AD compared with controls have been consistent. The possible influence of an overnight fast or food intake on A*β*_42_ levels has been brought forward mainly due to inconsistencies in studies concerning the plasma levels of A*β* [48]. However, in this study there was no significant difference between the baseline plasma A*β*_42_ level compared with either fasting or postprandial levels. Furthermore, it would thus seem unlikely that the CSF A*β* levels would be affected when the plasma levels were not.

### 3.6. Diurnal Variability

Diurnal variability in CSF A*β* levels would give cause for a standardized sampling time for everyday clinical routine. In a previous study, wherein 6 mL of CSF was withdrawn each hour, it was shown that A*β* had a large diurnal variability [50]. During a time period of 36 hours, the A*β* levels peaked at 12 hours and 23 hours with troughs at baseline and 25 hours with significant fluctuations of more than 50% within 6 hours. However, no complete return to baseline values was seen for A*β*. Our data showed more stable levels with a slight but significant decrease of 9.3% (*P* < .001) in CSF A*β*_42_ after 4–6 hours (mean 5.3 hours), which tended to return to baseline levels after 24 hours (4.4% lower than baseline; *P* = .002). 

 In this study, our attempt was to reflect the variation in CSF withdrawal time that might be a reality in some clinical settings. One explanation to the difference between our results and the study by Bateman et al. [50] might be that a smaller CSF volume was taken, which may cause less effect on the CSF dynamics. Even though the CSF A*β*_42_ level does not seem to be influenced by circadian rhythms to any greater extent, other analytes might be which would support a standardized time interval during the day for CSF withdrawal.

### 3.7. Centrifugation and Heat Denaturation

**Figure 3:**
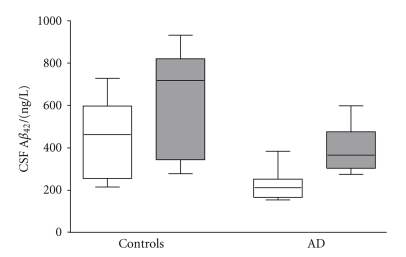
Boxplot (whiskers represent minutes and max values) of measured concentrations of A*β*_42_ in untreated and heat denatured CSF. The white boxes represent untreated CSF and dark boxes represent heat denatured CSF. Each box represents 15 samples (15 AD or 15 controls).

Ten samples were divided into three aliquots in order to test for precipitation during centrifugation, with and without cooling during the process. There was a significant (*P* = .002) decrease in the concentration of A*β*_42_ in both of the centrifuged samples (RT 203 ng/L (138–340); +4°C 203 ng/L (139–341)) when compared with the noncentrifuged samples (228 ng/L (147–354)). This indicates that a portion of A*β*_42_ in CSF might originate from cells that have undergone lysis that would precipitate together with the cells during the process of centrifugation. Furthermore, the A*β* fraction accessible to the antibody was further addressed by exposing CSF to heat denaturation. Herein, 18 CSF samples were divided into one heat exposed aliquot versus one unexposed aliquot and both were submitted to centrifugation prior to analysis. The A*β*_42_ concentration increased significantly (*P* < .001) in the heat treated samples (278 ng/L (170–471)) as compared with the untreated samples (210 ng/L (127–357)). This result was replicated in a case control study were the increase of A*β*_42_ was larger in the AD patient group (71%, *P* < .001) compared with the control group (42%, *P* < .001) (Figure 3). Consequently, the ROC analysis revealed a decreased discriminating power between AD and controls after heat treatment (AUC = 0.796) compared with the untreated samples (AUC = 0.907). The correlation for both test samples was high (*r*_*s*_ > 0.8, *P* < .001), when comparing untreated versus treated samples, indicating methodological stability.

### 3.8. Sample Pretreatment Affecting Assay Analysis

Factors known to affect the solubility and stability of proteins were investigated for its cofounding effects during analysis. Different buffer concentrations (10, 50 and 100 mM PBS, pH 7.4) and different buffer substances (PBS, Tris and HEPES, pH 7.4) did not affect the CSF A*β*_42_ measurement performance compared with the provided assay buffer. The pH of CSF was investigated and found to increase rapidly in RT from a starting value of pH 7.9 and reaching a plateau at pH 8.7 already after five hours. To test if the A*β*_42_ antibody binding capacity is altered due to differences in pH during analysis, which could be a confounding factor if employing a buffer with a low buffer capacity, pooled CSF was tested in three different pH systems (50 mM Tris, pH 7.4, 8 and 9). Compared with the levels of A*β*_42_ obtained in provided assay buffer system, the different pH tested did not alter the A*β*_42_ levels. Furthermore, BSA (0.5 mg/mL) and two different detergents (0.05% Triton100 and 0.1% Tween20) were added to test if the signal of A*β*_42_ could be improved by possibly decreasing the negative effects of protein interaction with the solid surface of the beads and/or the air-liquid interface. The signal was equally improved for all three additives (data not shown) as compared with the assay buffer system and the detergent effect was further assessed, what follows.

### 3.9. Epitope Masking and/or Assay Specific Variability

One hypothesis for the decreased level of A*β*_42_ in CSF from AD patients is that plaques in the brain act as sinks for A*β*_42_, preventing it from reaching the CSF. In CSF A*β* may either exist as a free soluble peptide, as oligomers [51], or bound in complex with carrier proteins such as *α*-2-macroglobulin [52], apolipoprotein E (ApoE) [53], apolipoprotein J (ApoJ; Clusterin) [54, 55], albumin [44], low-density lipoprotein receptor-related protein-1 (LRP) [56], and transthyretin [57]. The APOE *ε*4 allele is the strongest known genetic risk factor for AD [58, 59]. Carrier proteins such as ApoE are thought to play a part in the A*β* clearance and an altered clearance effect, in this case, is thought to be allele specific due to a decreased binding efficiency between A*β* and ApoE4 as compared with the other isoforms [60]. 

**Figure 4:**
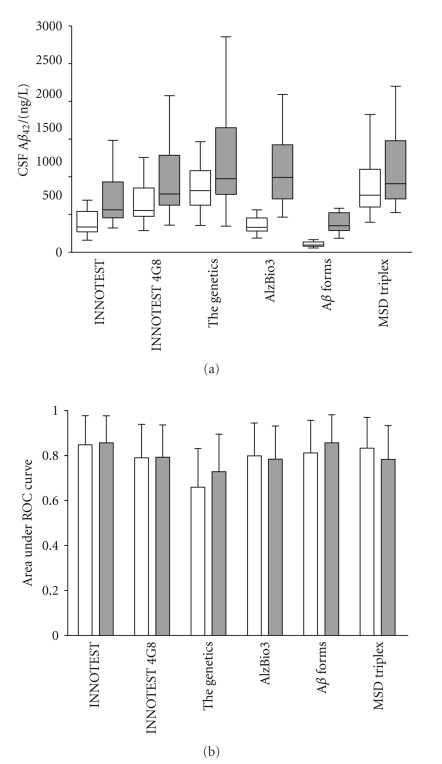
(a) Boxplot (whiskers represent minutes and max values) of CSF A*β*_42_ concentrations using different assays. For each kit the CSF was analyzed twice, neat and threefold diluted in a detergent-containing buffer. Each box represents 40 samples (20 AD + 20 controls). (b) Area under ROC curve for different assays with whiskers representing a 95% confidence interval. Neat, and threefold diluted CSF are represented by white and dark boxes/bars, respectively.

 CSF samples were treated with either detergent or heat denaturation, which has previously been shown to increase the A*β*_42_ measureable level [61, 62], to assess the fraction of possibly bound/epitope masked A*β*_42_ in proportion to the free A*β*_42_ in untreated samples and whether the total amount of A*β*_42_ could further improve the differentiation between AD and controls. The measured concentration of A*β*_42_ increased after the threefold dilution with the detergent containing buffer. The most striking increases were found for the xMAP assays AlzBio3 and A*β* forms. The A*β*_42_ median concentration for the neat CSF samples varied by more than a factor of eight, between the different assays tested (Figure 4(a)). The variation in the median is still present in the diluted samples but markedly reduced to less than a factor of three. To further investigate the result, the correlations between the assays were calculated (Table 2). Almost all tests, both neat and diluted, resulted in a difference in A*β*_42_ concentrations between AD and controls with *P* < .005. Only the ELISA assay from The Genetics failed to reach significance at this level with *P* = .086 and *P* = .017 for neat and diluted CSF, respectively. 

 The area under the ROC curve was used as a measure of the discrimination power for the assays (Figure 4(b)). Most of the assays performed equally well in discriminating between AD patients and healthy controls and there were no specific trend in the performance when the CSF was threefold diluted. Even though there are large differences in A*β*_42_ concentration depending on which assay is used most of the correlations between the assays are strong which indicates that the differences in measured concentrations are not due to cross reactivity for other substances than A*β*_42_. One possible explanation for the variation is that kit manufacturers have different sources for the A*β*_42_ standard that is used for calibration. This result highlights the need for an external A*β*_42_ control program that would allow manufacturers to calibrate their assays towards a common standard. The reason for the increase in measured concentration of A*β*_42_ upon dilution is at present unknown but might involve dissociation of A*β* homo- or heterocomplexes which would liberate more A*β*_42_ that are otherwise masked for detection. If this is true the results from the diluted samples would more truly reflect the total A*β*_42_ concentration, which potentially could be an even better biomarker than the “free” A*β*_42_ measured in the undiluted samples. However, there were no dramatic changes in the discriminating power in the diluted CSF compared with neat samples. Besides, methodological reasons for the increase cannot be excluded since the most dramatic changes are for the two assays based on the *x*MAP technology (Innogenetics' AlzBio3 and A*β* forms). Herein, it is clearly shown that divergences in absolute A*β*_42_ levels between different centers could be explained by the fact that different ELISAs are utilized with different protocols as well as assay methodologies (Table 1). However, when different centers employ the same ELISA from the same manufacturer divergences often still remain [63]. Another factor affecting the A*β* concentration inconsistency might be the result of a lot-to-lot variability [64]. 

 Detergent and heat treatments give rise to a similar increase in the measured level of A*β*_42_ in the AD groups, 75% and 71%, respectively. In contrast, the detergent treated control CSF diverged from the heat denatured by a more pronounced increase (83% versus 42%). Whether the divergences in the increase of A*β*_42_ levels between the two differently treated CSF samples of the controls and the discriminating power between neat/detergent CSF compared with native/denatured samples could be explained by the differences in study sample, methodological reasons or differences in complex stability needs to be addressed by further studies.

## 4. Conclusion

Due to the high between-center variability (possibly caused by preanalytical and analytical factors) of reported A*β*_42_ levels in CSF, possible confounding factors were assessed in relation to the CSF A*β*_42_ levels. The confounding factors found to influence the preanalytical procedures for CSF collection and sample processing, analytical procedures and techniques ultimately leading to altered A*β*_42_ concentrations are summarized below.

**Preanalytical Factors**

(i) Increased A*β*_42_ concentration in noncentrifuged CSF samples possibly due to a release of the analyte caused by cell lysis—it is important to centrifuged CSF within a standardized time interval after LP.

(ii) Decreased A*β*_42_ levels due to adsorption of analyte to different types of test tubes—standardization of test tubes used for CSF sampling that is, polypropylene.

(iii) Pretreatment of CSF with detergent-containing buffers or heat denaturation lead to an increase in A*β*_42_ levels—probably due to dissociation of A*β* bound to proteins or release of A*β* from oligomers. For these reasons a standardization of dilution factors, buffer additives and sample processing is necessary prior to analysis.

(iv) The CSF A*β*_42_ concentration decreased at the addition of plasma corresponding to a CSF/serum albumin ratio of 11–55—probably due to the binding of free A*β* to plasma proteins.

**Analytical Factors**

(i) Different immuno-assays employing various antibodies and possibly dissimilar sources for the calibrator peptides lead to divergences in the absolute A*β*_42_ concentration—between center comparisons cannot be made when employing different assays. This problem cannot be solved until an international A*β* golden standard is available.

Even though the CSF concentration of A*β*_42_ does not seem to be affected by a spinal chord gradient, circadian rhythms, blood contamination or by storage/thawing conditions other proteins may be. It is necessary to use a standardized protocol to allow for between-center comparisons, for a detailed protocol see Blennow et al. [9].

##  Disclosure 

K. Blennow has participated in an advisory board for the Innogenetics. The other authors have nothing to disclose.

## Figures and Tables

**Table 1:** Properties of evaluated commercially available A*β* assays. The various capture and detection antibodies employed in the different A*β*_42_ immunoassays. The differences in antibody epitope recognition and the part of CSF diluted in buffer during the incubation render methodological differences both qualitatively and quantitatively. aa, amino acids.

	Innotest Elisa	Innotest Elisa (4G8)	The genetics	INNO-BIAAlzBio3	INNO-BIAA*β* forms	MSD Triplex
Capture (epitope)	21F12 (42 C-terminal)	21F12 (42 C-terminal)	W02 (aa 5–8)	4D7A3 (42 C-terminal)	21F12 (42 C-terminal)	Not declared (42 C-terminal)
Detection (epitope)	3D6 (aa 1–5)	4G8 (aa 17–24)	G2-13 (42 C-terminal)	3D6 (aa 1–5)	3D6 (aa 1–5)	4G8 (aa 17–24)
CSF (% v/v)	25	25	50	75	75	50
A*β*	1–42	x-42	x-42	1–42	1–42	x-42

**Table 2:** Correlation matrix for the evaluated A*β* assays. The correlation between the levels of A*β*_42_ in different assays. The correlation coefficients for the neat and diluted CSF samples are shown in the upper right and lower left part, respectively.

	Innogenetics	Innogenetics	The Genetics	Innogenetics	Innogenetics	MSD
	ELISA (4G8)	ELISA	ELISA	AlzBio3	A*β* forms	Triplex
Innogenetics ELISA (4G8)	1	0.94	0.90	0.87	0.69	0.97
Innogenetics ELISA	0.96	1	0.82	0.93	0.81	0.92
The Genetics ELISA	0.93	0.88	1	0.75	0.53	0.87
Innogenetics AlzBio3	0.92	0.94	0.89	1	0.88	0.84
Innogenetics A*β* forms	0.78	0.86	0.67	0.86	1	0.66
MSD Triplex	0.98	0.96	0.93	0.93	0.77	1
